# Duck pan‐genome reveals two transposon insertions caused bodyweight enlarging and white plumage phenotype formation during evolution

**DOI:** 10.1002/imt2.154

**Published:** 2023-12-17

**Authors:** Kejun Wang, Guoying Hua, Jingyi Li, Yu Yang, Chenxi Zhang, Lan Yang, Xiaoyu Hu, Armin Scheben, Yanan Wu, Ping Gong, Shuangjie Zhang, Yanfeng Fan, Tao Zeng, Lizhi Lu, Yanzhang Gong, Ruirui Jiang, Guirong Sun, Yadong Tian, Xiangtao Kang, Haifei Hu, Wenting Li

**Affiliations:** ^1^ Henan Key Laboratory for Innovation and Utilization of Chicken Germplasm Resources, Department of Animal Genetic and Breeding, College of Animal Science and Technology Henan Agricultural University Zhengzhou China; ^2^ The Shennong Laboratory Zhengzhou China; ^3^ Shenzhen Branch, Guangdong Laboratory for Lingnan Modern Agriculture, Agricultural Genomics Institute at Shenzhen Chinese Academy of Agricultural Sciences Shenzhen China; ^4^ Key Laboratory of Agricultural Animal Genetics, Breeding and Reproduction of Ministry of Education, Intelligent Husbandry Department, College of Animal Science and Technology Huazhong Agricultural University Wuhan China; ^5^ Wuhan Academy of Agricultural Science Wuhan China; ^6^ Simons Center for Quantitative Biology Cold Spring Harbor Laboratory Cold Spring Harbor New York USA; ^7^ Department of preventive veterinary medicine, College of Veterinary Medicine Henan Agricultural University Zhengzhou China; ^8^ International Joint Research Center for National Animal Immunology Zhengzhou Henan China; ^9^ Quality Safety and Processing Laboratory Jiangsu Institute of Poultry Sciences Yangzhou China; ^10^ State Key Laboratory for Managing Biotic and Chemical Threats to the Quality and Safety of Agro‐Products, Institute of Animal Husbandry and Veterinary Science Zhejiang Academy of Agricultural Sciences Hangzhou China; ^11^ Rice Research Institute, Guangdong Key Laboratory of New Technology in Rice Breeding and Guangdong Rice Engineering Laboratory Guangdong Academy of Agricultural Sciences Guangzhou China

**Keywords:** *IGF2BP1*, *MITF*, pan‐genome, structural variation, transposable element

## Abstract

Structural variations (SVs) are a major source of domestication and improvement traits. We present the first duck pan‐genome constructed using five genome assemblies capturing ∼40.98 Mb new sequences. This pan‐genome together with high‐depth sequencing data (∼46.5×) identified 101,041 SVs, of which substantial proportions were derived from transposable element (TE) activity. Many TE‐derived SVs anchoring in a gene body or regulatory region are linked to duck's domestication and improvement. By combining quantitative genetics with molecular experiments, we, for the first time, unraveled a 6945 bp Gypsy insertion as a functional mutation of the major gene *IGF2BP1* associated with duck bodyweight. This Gypsy insertion, to our knowledge, explains the largest effect on bodyweight among avian species (27.61% of phenotypic variation). In addition, we also examined another 6634 bp Gypsy insertion in *MITF* intron, which triggers a novel transcript of *MITF*, thereby contributing to the development of white plumage. Our findings highlight the importance of using a pan‐genome as a reference in genomics studies and illuminate the impact of transposons in trait formation and livestock breeding.

## Highlights

- We present the first duck pan‐genome constructed using five genome assemblies capturing ∼40.98 Mb new sequences absent from the reference genome.
- We find a significant portion of the detected structural variants were derived from transposable element (TE) activity. Many of these are located within gene bodies or regulatory regions, potentially linked to duck domestication and enhancement.
- We used two representative examples to show how TE insertions can lead phenotypic diversity, highlighting *IGF2BP1*'s role in bodyweight and *MITF*'s influence on white plumage in ducks.
- Notably, the Gypsy insertion in the *IGF2BP1* promoter, to our knowledge, explains the largest effect on bodyweight among avian species (27.61% of phenotypic variation).

## Graphical abstract

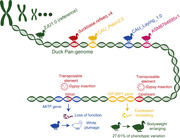

## INTRODUCTION

The duck (*Anas platyrhynchos*) is a major source of meat and eggs for human consumption and is also a significant source of downy feathers. Ducks were domesticated in approximately 500 BC in central China during the Iron Age, descending from wild mallards (*Anas platyrhynchos*) and spot‐billed duck (*Anas zonorhyncha*) [1, 2, 3]. There are 37 indigenous duck breeds in China, which are mainly distributed in the east, center, southwest, and south of China, with a concentration in the Yangtze River and the Pearl River basins regions. Evidence presented by Zhou et al. suggests that mallards took approximately 500–800 generations to domesticate into indigenous ducks, and indigenous ducks underwent approximately 700–1000 generations of breeding to develop into the commercial Pekin duck [4]. Pekin ducks were introduced to Cherry Valley Farm in England contributing to the breeding of Cherry valley ducks, and to France, where they contributed to the breeding of Grimaud Freres ducks. Domestication and subsequent breeding improvement have led to phenotypic variations in ducks, most notably in their morphology, productivity, and behavior [5].

Phenotypic variation in livestock is shaped by genetic variants accumulated during the domestication from wild ancestors to indigenous and modern breeds. Several duck phenotypes have been elucidated through single‐nucleotide polymorphism (SNP)‐based studies. For instance, two SNPs located in the regulatory region of *MC1R* are associated with black plumage in Mallard × Pekin F2 population [6]. An SNP mutation in the 5′ untranslated region of *TAS2R40* caused the crest formation in Chinese crested duck [7]. Additionally, two *cis*‐regulatory SNPs located upstream of *ABCG2* were involved in the blue eggshell of Jinding duck [8]. Nonetheless, many economically significant traits remain unresolved as the causal variant remains unidentified. For example, many SNPs located upstream of *IGF2BP1* are significantly associated with body weight. However, the causal variant is still unidentified.

Decades of research showed structural variations (SVs) are important in agriculture and evolution, affecting phenotypes including feathered legs, crest, and body size of chicken [9, 10, 11], as well as plumage pigmentation of pigeon [12]. Compared to SNP function, SVs can cause large‐scale perturbations of *cis*‐regulatory regions leading to quantitatively changing gene expression or novel transcript yielding to distinct phenotypes [13]. Previous studies also suggest that SV generation is associated with transposable elements (TEs) mediated imprecise duplication, insertion, deletion, and reshuffling of host genome sequences during genome evolution [14, 15, 16, 17]. In addition, TE‐derived SVs contribute to phenotypic variations in vertebrates by affecting the transcriptional regulation via donating promoter or enhancer sequences, modifying three‐dimensional chromatin architecture that regulates the host genes' expressions and leading to de novo gene birth [15, 18, 19]. Examples include henny feathering in chicken [20], white coat color in buffalo [21], secondary palate development [22], and embryonic implantation in humans [23]. However, a single reference genome derived from one individual cannot fully capture a species' genetic diversity, leaving the majority of SVs poorly resolved and their phenotypic impacts largely hidden during domestication and breeding improvement.

A pan‐genome, which captures the complete genetic variations of a species via integrating pan‐sequences from multiple genome assemblies, enables a comprehensive survey of SV landscapes with population‐scale sequencing data, resulting in better characterizations of SVs and the understanding of their impacts on phenotypic variations. Numerous animal pan‐genomic studies have demonstrated their capacity to facilitate and provide insights into the dissection of agronomic traits based on SVs in chicken [9], sheep [24], and cattle [25]. Here, we constructed the first duck pan‐genome using five genome assemblies and investigated SVs in 12 populations of 131 ducks (wild, native, and commercial breeds) with high‐depth sequencing data. By identifying the associations between SVs and TEs across the pan‐genome, we found an increase in the occurrence probability of SVs linked with TE insertions. To demonstrate the significance of these TE‐derived SVs, we linked these SVs with domestication and improvement traits. We found these SVs affected the expression level and contributed to new transcript generation of causative genes regulating the quantitative bodyweight trait and qualitative plumage pattern. Our work expands the understanding of the importance of the pan‐genome and underlines the pronounced effect of TE‐derived SVs on phenotype formation.

## RESULTS

### Construction of duck pan‐genome

**Figure 1:**
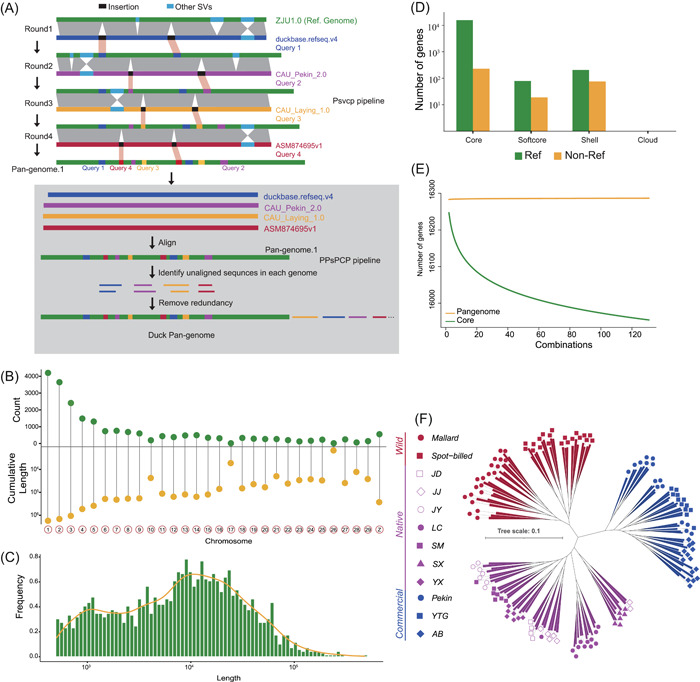
Pan‐genome of Duck. (A) Schematic of duck pan‐genome construction. (B) Statistics of newly identified sequences placed in chromosomes via *Psvcp* pipeline. (C) Length distribution of novel sequences identified via *PPsPCP* pipeline. (D) Classification of Pan‐genome genes. (E) Pan‐genome modeling. (F) Phylogenetic tree constructed based on SVs with GTR model. Wild: wild group, including mallard and spot‐billed (Chinese spot‐billed duck); native: indigenous duck breeds, including JD (Jinding duck), JJ (Jingjiang sheldrake), JY (Jinyun sheldrake), LC (Liancheng white), SM (Shan sheldrake), SX (Shaoxing), and YX (Youxian sheldrake) duck; commercial: commercial duck breeds, including Pekin, YTG (Cherry Valley), and AB (Grimaud freres) duck. SV, structural variation.

We constructed the first duck pan‐genome using a combination approach of *Psvcp* [26] and *PPsPCP* [27] pipelines (Materials and Methods). The five published duck genomes were used to construct the duck pan‐genome, which consists of three Pekin duck genomes (commercial breed), one Shaoxing duck genome (indigenous breed), and mallard duck genome (wild relatives) (Table S1). The approach we used for constructing the duck pan‐genome is visualized in Figure 1A. Briefly, duckbase. refseq.v4 genome was aligned to the initial reference genome (ZJU1.0), insertions longer than 50 bp were then identified and placed in the ZJU1.0. This process was further iterated by CAU_Pekin2.0, CAU_Laying_1.0, and ASM8764695v1 in an order of increasing phylogenetic distance to ZJU1.0 (Figure S1A), and thus the Pan‐genome.1 was generated. Subsequently, four query genomes were aligned to Pan‐genome.1, respectively, while novel contigs longer than 500 bp were retained after removing redundancy. Novel contigs and Pan‐genome.1 were merged into the final duck pan‐genome (Table S2). The duck pan‐genome identified ∼40.98 Mb additional sequences that were absent from the reference genome (ZJU1.0), encoding 329 high−confidence genes with intact coding regions. In the *Psvcp* pipeline, 21,403 insertions were identified and placed into 30 chromosomes, cumulatively encoding the genomic length of ∼7.42 Mb (Figure 1B). Novel contigs generated from the *PPsPCP* pipeline comprised 1830 sequences cumulatively encoding genomic sequence length of ∼33.56 Mb (Figure 1C).

We further generated 131 high‐depth sequencing data from 3 commercial duck breeds, 7 indigenous breeds, and 2 wild species, which represent the majority of the genetic diversity of ducks, with an estimated average depth of up to 46.5× through the pan‐genome (Table S3). We categorized genes in the duck pan‐genome according to their gene presence/absence variation (PAV) frequencies in all duck breeds. A total of 15,906 (97.67%) core genes were shared by 131 individuals. The remaining 380 (2.33%) were categorized as dispensable genes, including 98 softcore and 282 shell, defined by a presence frequency exceeding 99% and 1%–99% respectively (Figure 1D). This duck pan‐genome exhibits a higher proportion of core gene content compared to that of human (96.88%) [28], chicken (76.32%) [9], and mussel (69.2%) [29]. Gene Ontology (GO) analysis revealed that dispensable genes were enriched in terms including immune response, sensory perception of smell, and G protein‐coupled receptor signaling pathway (Figure S2). Evidence from pan‐genome modeling revealed a closed pan‐genome with an estimated total of 15,959 genes (genes on sex chromosomes were excluded, Figure 1E), suggesting the current assembled pan‐genome included nearly complete genetic diversity of ducks.

### Identification of population‐wide genetic variations

Four different SV detection tools LUMPY [30], Delly [31], GRIDSS [32], and Manta [33] commonly used for SV detection in animals [34] and plants [35] were employed. SVs were identified based on our pan‐genome using high‐depth sequencing data (average 46.5×) with support by at least two SV detection tools to minimize false positive SV discoveries. As a result, we generated a final set of 101,041 SVs among 131 duck genomes. Due to the inherent limitations of short‐read sequencing and its associated SV detection algorithms, biases in SV discovery may occur [36]. To estimate the accuracy of SV detection in this study, we validated the consistency of four randomly selected SVs detected at the population level using polymerase chain reaction (PCR) genotyping. Based on the PCR results of 131 samples × 4 long fragment SVs genotypes, the average accuracy rate is 87.4% (Table S4), similar to the 88% SV calling accuracy reported in a peach SV discovery study [35]. To further demonstrate the advantage of using a pan‐genome in the genetic variant detection, we found that using the pan‐genome as the reference can lead to the identification of an additional 27,533 autosomal SVs and 3,155,258 small genetic variants including 2,912,903 SNPs and 242,335 indels compared to the single reference genome ZJU1.0. The SV length distribution evaluation revealed that most SVs are shorter than 1000 bp (Figure S3A). It is important to note that our short‐read sequencing‐based SV discovery methods are limited by read length and thus often unable to identify larger insertions. SV‐based genetic analysis revealed that 131 individuals clustered into three major groups: wild, native, and commercial, as shown in the phylogenetic tree, principal component analysis and population structure (Figure 1F, Figure S3B,C). Evolutionary relationships inferred from SVs are mostly consistent with the evidence inferred from SNPs (Figure S4).

### Change of SV frequency during duck domestication and improvement

**Figure 2:**
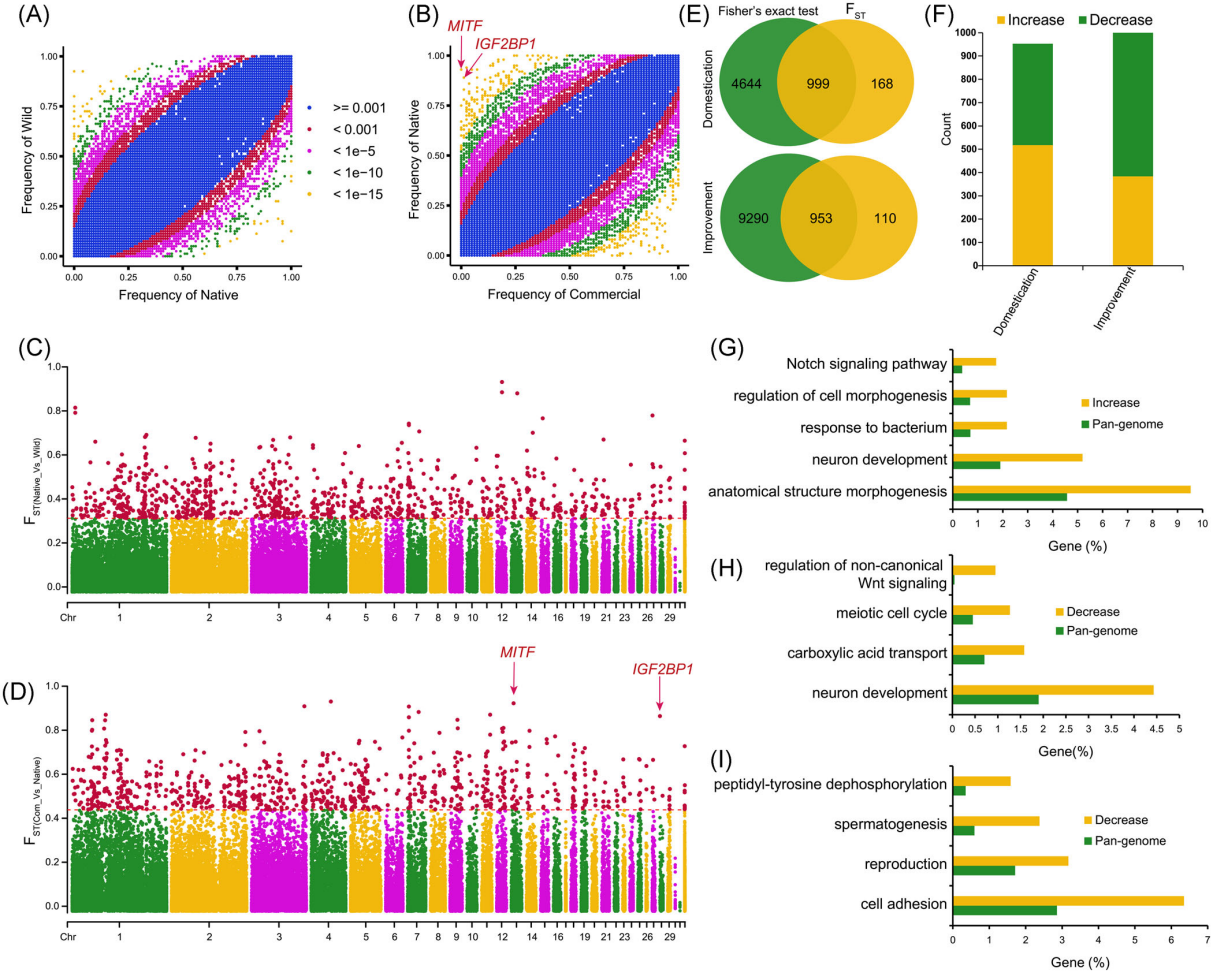
Selection of SVs during duck domestication and improvement. Scatter plots showing SV occurrence frequencies in (A) wild and native (comparisons for domestication) and in (B) native and commercial groups (comparisons for improvement). Manhattan plots showing *F*_ST_ between (C) native and wild groups, and between (D) native and commercial groups. Top 1% was defined as significant SVs. (E) Venn plot showing the overlap between Fisher's exact and *F*_ST_ significant SVs. (F) Classification of significant SVs during evolution. Enriched GO terms in genes closest to (G) frequency increased and (H) decreased SVs during domestication, and (I) decreased SVs during improvement. GO, Gene Ontology; SV, structural variation.

To uncover the changes in SV occurrence frequencies during duck domestication and subsequent breeding improvement, we conducted two sets of comparisons between the wild and native breeds for domestication (Figure 2A,C) and between the native and commercial breeds for breeding improvement (Figure 2B,D). The overlap of significant results between a Fisher's exact test [9, 37] and the Fixation index (*F*_ST_) value [25] was defined as the SVs selected during domestication or improvement. We observed occurrence frequencies of 999 SVs showing significant differences between native ducks and wild ducks, with 382 SVs increased and 617 SVs decreased in frequencies (Figure 2E,F and Table S5). GO analysis indicates that genes adjacent to SVs selected regions (potentially selected genes) during domestication were enriched in functions associated with neuron development, anatomical structure morphogenesis, cell morphogenesis, response to bacterium process, and so forth (Figure 2G,H). Due to captivity and selection during domestication [38], a 24%–35% reduction in brain size was reported in ducks [39, 40], leading to neuron development alteration of visual and trigeminal systems in the telencephalon. Potentially selected genes enriched in structure morphogenesis and cell morphogenesis may contribute to the increase in body size of native ducks during domestication [4]. Potentially selected genes involved in response to bacteria may result from living environment alterations and changes in pathogen pressure during domestication. In addition, one promising genes *ELOVL3* was potentially selected during domestication (Figure S4E). The *ELOVL3* gene is essential for unsaturated fatty acid metabolism, which might explain the observed lower content of lower long‐chain fatty acid in the yolks of eggs from captive birds, including duck, compared to their wild counterparts [41].

Besides, 518 SVs increased and 435 SVs decreased in frequencies were detected between commercial ducks and native ducks (Figure 2E,F and Table S6). Genes affected by these selected SV during improvement were enriched in cell adhesion, reproduction, spermatogenesis, peptidyl‐tyrosine dephosphorylation (Figure 2I). The fact that reproduction associated genes were under further selection during breeding improvement is in line with higher performance of egg production in commercial breeds compared with native ducks. Another potentially selected gene, fibronectin 1, could enhance chondrocyte differentiation and collagen production, which are essential for duck sternal ossification [42]. This gene may also participate in the developmental trade‐off prompted by artificial selection, balancing an increase in breast muscle yield with incomplete sternal ossification and a slower growth rate in the sternum area [43]. Intriguingly, we note that two well‐studied genes associated with productive and morphological traits, respectively, were adjacent to deletions with significant frequency changes during improvement (Figure 2B). A ∼7.0 kb deletion (ID: DEL00154411) located at the upstream region of *IGF2BP1* shows a higher occurrence frequency (0.89) in native ducks compared with an extremely lower frequency (0.012) in commercial ducks (false discovery rate [FDR]‐adjusted *p* = 5.54*e*−34 and *F*_ST_ = 0.86). Another ∼6.6 kb deletion (ID: DEL00130156) located in an intron of *MITF* presents a significantly higher frequency (0.93) in native ducks while completely absent in commercial ducks (FDR‐adjusted *p* = 4.55e−39 and *F*_ST_ = 0.92).

### Transposon‐derived SVs are linked to duck domestication and improvement

**Figure 3:**
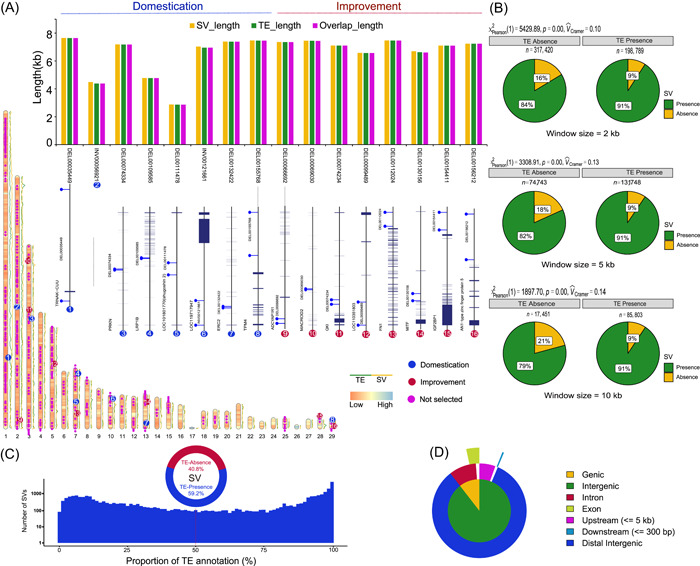
Transposable elements (TEs) annotation and their association with SVs based on duck pan‐genome. (A) Localizations of SVs and TEs across the duck pan‐genome. Alone with the schematic chromosomes, the green line represents the TE count of each Mb window; the yellow line represents the SV count of each Mb window; the purple dots above the schematic chromosomes show the position of intact‐TE derived SVs, while red and blue dots represent the selected SVs during domestication and improvement, respectively. The embedded histogram shows the length of SV, TE, and the overlap length between intact‐TE and matched SVs under selection; positional relationships between 15 intact‐TE derived SVs and their nearest genes were labeled below, which SVs located between two blue sticks. (B) Co‐occurrence probability between SVs and TEs across the pan‐genome investigated by the *χ* ^2^ tests. (C) The proportions of SVs matched to TEs and the distribution of overlap proportions with TE (below). (D) Percentage of genomic features with high TE‐derived SVs. SV, structural variation.

Using de novo‐prediction and sequence similarity detection methods, around 24.37% of the newly identified sequences were annotated as TEs, while only 9.49% of entire duck pan‐genome sequences were TEs. Accumulative evidence revealed that TEs offer the fodder for pan‐genome dynamics [44, 45]. TEs transposition propagation experienced insertion and removal processes that easily lead to SV generation because of imprecise manipulation, reshuffling of host genome sequences, and recombining of highly homologous regions [15, 16, 17]. These factors implied the possibility of SV generation correlated to TE distribution during genome evolution. Therefore, we further investigated the classification, abundance, and length of TEs across the pan‐genome (Figure 3A, Figure S5) and estimated the presence correlation between TEs and SVs using a sliding window approach with different window sizes (Figure 3B). Significant presence correlations between SVs and TEs were observed for all the tested window sizes (Figure 3B). The presence frequency of SVs significantly increased when TEs were present. We observed that 59.2% of identified SVs larger than 100 bp correspond to at least one TE, of which 58.7% of these identified SVs show at least half of their sequence overlapping with TEs (SVs with high TE overlap) (Figure 3C). SVs with high TE overlap are comprised of 42.24% deletions, 55.86% inversion, and 0.90% duplication, respectively. Among these, 10.48% were located within a gene body and 10.79% were located in the 5 kb upstream region of a gene (Figure 3D). This implies that SV generation may be correlated to TE distribution and has played a role in regulating gene function during duck genome evolution.

To reveal which SVs were entirely derived from intact TEs insertion across the duck pan‐genome, 5240 intact TEs were identified using the extensive de‐novo TE annotator (EDTA) pipeline, and 392 SVs were matched to identified TEs with sequence overlap of more than 95% (Table S7). Of these intact TE‐derived SVs, we found that the occurrence frequencies of 16 SVs were significantly altered during the evolution of domesticated ducks, including 8 SVs during domestication and 8 SVs during improvement (Figure 3A). Of these 16 SVs, 8 located within the intron of genes and 7 were found in the upstream or downstream (from 0.88 to 492.5 kb) of genes (Figure 3A). Interestingly, these intact TE‐derived SVs included the two involving well‐studied genes, as mentioned above: DEL00154411 locates upstream of *IGF2BP1* and DEL00130156 within the intron of *MITF*. SVs triggered by TEs could alter the expressions of the nearest genes or generate novel transcripts of host genes, which is likely to diversify the phenotypes, including quantitative and qualitative traits. Subsequently, we took the SVs in *IGF2BP1* and *MITF* as examples to decipher how TE‐derived SVs can drive duck phenotypic evolution.

### Insertion of a 6945 bp Gypsy transposon into the promoter region of *IGF2BP1* increases duck bodyweight via higher *IGF2BP1* expression

**Figure 4:**
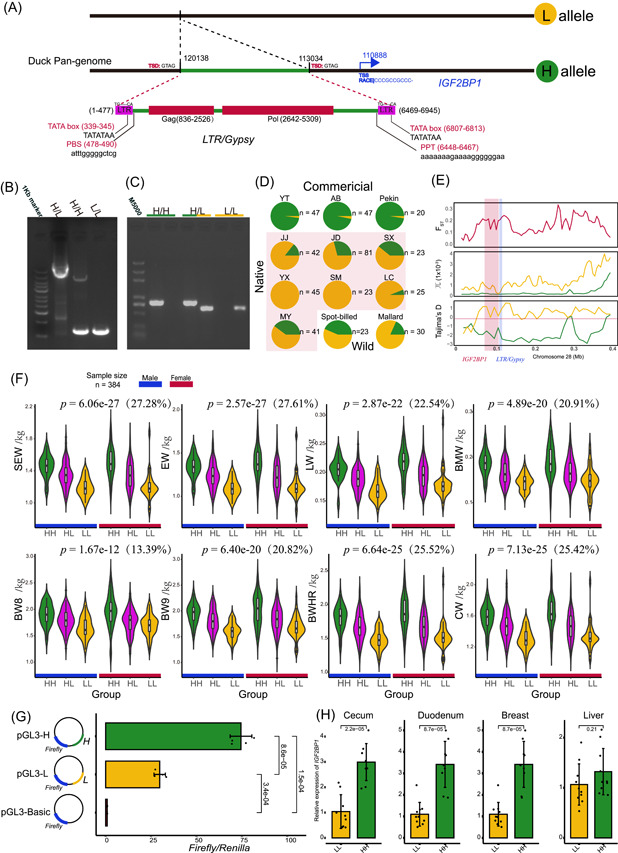
The Gypsy element regulated *IGF2BP1* expression and was associated with bodyweight traits. (A) The structure of the Gypsy element is anchored in the *IGF2BP1* promoter region. (B) Gel plots for the PCR genotyping and (C) allelic‐specific PCR genotyping. (D) Allelic frequencies of the *IGF2BP1* promoter insertion in the validated populations by allelic‐specific PCR genotyping. (E) SNP‐based selective sweep analysis on Gypsy element between commercial (green line) and native (yellow line) group. (F) Single‐marker genotype association of *IGF2BP1* promoter insertion in validated duck populations. The percentage in the brackets is the proportion of phenotype variance explained by the insertion. (G) Comparison of transcriptional activity between pGL3‐L and pGL3‐H recombinant plasmids representing different genotypes of *IGF2BP1* regulatory region. The significance level was analyzed by a two‐tailed Student's *t* test. (H) Comparison of mRNA expression of *IGF2BP1* between HH (YTG) and LL (SM) ducks in four tissues at 1 day of age. *p* values were calculated using a two‐tailed Student's *t* test. BMW, breast muscle weight, BW8 and BW9, bodyweight at the age of eighth and ninth week; BWHR, bodyweight at slaughter; CW, carcass weight; EW, evisceration weight; LW, leg weight; mRNA, messenger RNA; PCR, polymerase chain reaction; SEW, semievisceration weight.

*IGF2BP1* was reported to be associated with the bodyweight of duck, in which a higher level of messenger RNA (mRNA) is correlated to a higher bodyweight [4]; however, the causative variant of *IGF2BP1* responsible for bodyweight variability remains unresolved. Our pan‐genome analysis and subsequent PCR Sanger‐sequencing revealed a 6945 bp genome sequence insertion in the *IGF2BP1* promoter region, the presence and absence of which define H (heavy) and L (light) alleles of the gene, respectively (Figure 4A,B, and Figure S6A). The result from allele‐specific PCR demonstrated that the H allele is nearly fixed in the commercial breeds. In contrast, the L allele is dominant in the native and wild breeds (Figure 4C,D, Figure S6B), consistent with the increase in the frequency of this SV during improvement (Figure 2). Evolutionary analysis demonstrated that the 6945 insertion was under selected during improvement rather than genetic drift (Figure 4E). Given that the 6945 bp insertion could be a marker linked to the causal variant, we further investigated the genetic polymorphisms within 20 kb genomic region flanking the 6945 bp inserted sequence. We calculated the absolute allelic frequency differences (△AF) between individuals from commercial duck breeds with higher bodyweight and those with lower bodyweight. Of the 90 investigated genetic variants, the 6945 bp insertion displayed the highest △AF (0.90). This suggests that this insertion located in the promoter region of *IGF2BP1*, is the top candidate variant responsible for the heavier body weight in duck.

Single‐marker association analysis using a Liancheng white duck × Kaiya duck F2 population demonstrated that this SV is significantly associated with eight bodyweight and carcass weight (CW) traits which include evisceration weight (EW), semievisceration weight (SEW), leg weight (LW), breast muscle weight (BMW), bodyweight at the age of eighth and ninth weeks (BW8 and BW9), bodyweight of feather removed at slaughter (BWHR), and CW (Figure 4F). Regarding these traits, the HH genotype was always linked to a higher performance of production compared to the LL genotype. Of these, associations are most significant in EW (*p* = 2.57*e*−27) and SEW trait (*p* = 6.06*e*−27), and this locus accounts for 27.61% and 27.28% of phenotypic variations, respectively. The other traits affected by the HH genotype also presented a strong effect, with more than 20% of phenotypic variation explained for all other traits except BW8 (Figure 4E). For BW8, HH explained 13.39% of phenotypic variation.

The 6945 bp insertion was predicted to be an intact TE‐derived SV. As a result, this TE‐derived SV belongs to the *Gypsy* long terminal repeat (LTR) superfamily with two intact LTRs as well as *gag* and *pol* coding genes (Figure 4A). Retrotransposons can alter host gene expression or generate novel fusion transcripts [18]. Our quantitative PCR data showed that mRNA expression of the ducks with *IGF2BP1* HH genotype (YTG) was significantly higher than those ducks with the LL genotype (SM) in nearly all examined tissues at Day 1 of age (Figure 4H). Additionally, RNA sequencing (RNA‐seq) analysis further confirmed this result, by showing that the expression level of *IGF2BP1* in the LL genotype (mallard) was lower than that in the HH genotype (Pekin) across the liver and sebum tissues at 2, 4, and 6 weeks of age (Figure S6C). After we investigated *IGF2BP1* transcription, evidence from the rapid amplification of complementary DNA (cDNA) ends (RACE) shows that no fusion transcript of *IGF2BP1* was generated and the transcription start site (TSS) was positioned at Chr28:110,888 (Duck pan‐genome). This suggests that, although the 3′LTR of this *Gypsy* contains a TATA box, rather than a promoter, it likely acts as an enhancer regulating the gene ∼2 kb away (Figure 4A). To verify the effect of this TE on transcriptional activity, two recombinant plasmids pGL3‐H and pGL3‐L were constructed, representing the upstream regions of *IGF2BP1* including possible promoters/enhancers for mutant and wild‐type alleles, respectively. Before performing the luciferase activity experiment, we screened the inserted genomic sequence between pGL3‐H and pGL3‐L, and confirmed that we did not find any difference except the 6,945 insertion. The transcriptional activity of pGL3‐H was significantly higher than that of pGL3‐L (Figure 4G). By combining predicted binding transcription factors (TFs) and RNA‐Seq detected regulatory factors, only one binding site with binding scores greater than 10 was identified (SOX6 or SOX9 binding to the GCATTGTTTG sequence, from 6722 to 6731 bp of the *Gypsy* insertion). Based on the above evidence, we conclude that a 6945 bp Gypsy element inserted into the promoter region of *IGF2BP1* might increase the duck bodyweight by providing an extra enhancer element to increase *IGF2BP1* expression.

### A 6634 bp Gypsy element inserted into the intron of *MITF* generates a chimeric transcript that contributes to the white plumage phenotype in duck

**Figure 5:**
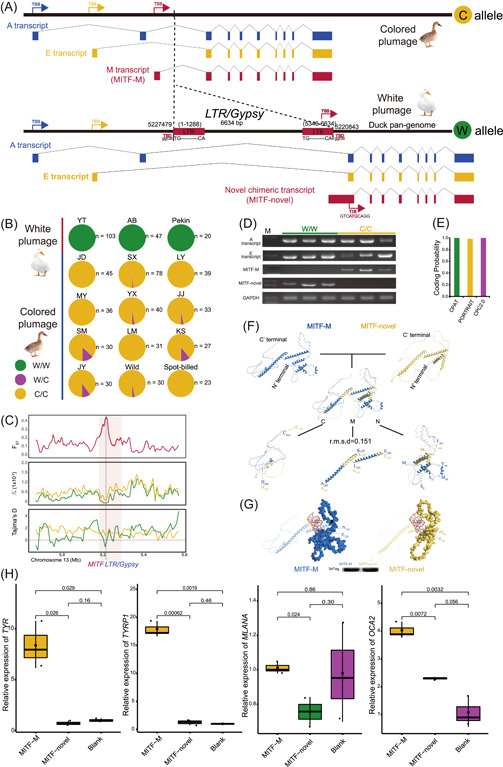
The novel chimeric transcript of *MITF* induced by a Gypsy insertion and its function. (A) The structure of the Gypsy that is anchored in the intron of *MITF* and identification of its transcript structure. (B) Genotypic frequencies of the *MITF* intron insertion in the validated populations with white and colored plumage by allelic‐specific PCR genotyping. (C) SNP‐based selective sweep analysis on Gypsy element between white plumage (green line) and color plumage (yellow line) group. (D) Gel plots for the validation of identified transcripts using PCR amplification. (E) The Coding probability analysis for the MITF‐novel transcript. (F) The overall structure and alignment of MITF‐M and MITF‐novel in cartoon mode. M represents the middle region of proteins, N represents the N‐terminal region, and C represents the C‐terminal region. Western blot showing the coding ability of MITF‐M and MITF‐novel using 3× FLAG antibody. (G) Amino acid binding characteristics of MITF‐M and MITF‐novel. The N‐terminal regions show surface mode, and the rest show cartoon mode. The orange spiral structure indicates DNA fragments. (H) Quantitative PCR results showing the relative expression levels of four downstream genes of *MITF* in *MITF*‐M overexpression cells MITF‐novel overexpression cells and blank. PCR, polymerase chain reaction; SNP, single nucleotide polymorphism; TIS, translation initiation site; TSS, transcriptional start site.

Previous studies showed that a ∼6.6 kb insertion in the intron of *MITF* was associated with the white plumage phenotype of Pekin and Cherry Valley duck [4, 46]. However, the molecular mechanism of this insertion remains unknown. Our pan‐genome analysis and subsequent PCR Sanger‐sequencing identified this insertion's detailed location and sequence, which is 6634 bp in length. The presence and absence of this insertion are defined as W (white) and C (colored) alleles, respectively (Figure 5A). Results from the allele‐specific PCR demonstrate that the WW genotype is completely absent in ducks with colored plumage while completely fixed in commercial white plumage ducks (Pekin, YTG, and AB) (Figure 5B, Figure S7A–D). WW homozygote is linked to the white plumage phenotype, while WC heterozygote and CC homozygote present colored plumage, suggesting the W allele is recessive relative to the C allele. Evolutionary analysis demonstrated that the 6634 insertion was under selected rather than genetic drift (Figure 5C).

The insertion of the W allele is predicted to be a member of the Gypsy superfamily, encoding two intact LTRs. However, intact proviral elements were not identified (Figure 5A). This may be due to the accumulation of mutations following the TE insertion. Based on the sequence homology of the LTR region, this insertion occurred ∼0.6 million years ago, assuming the substitution rate of 1.91 × 10^−9^ per site per year [47]. As intact proviral elements were absent, we investigated whether the intact LTR region might cause the effect of the W allele on *MITF*. We conducted RACE to identify the *MITF* transcripts in skin tissue from ducks at 1 day of age. In the SM duck with *MITF* CC genotype, three kinds of transcripts of *MITF* were identified, consisting of A, E, and MITF‐M (Figure 5A,D). The MITF‐M transcript of *MITF* is expressed exclusively in melanocyte lineages regulating the expressions of numerous pigmentation genes in the melanogenesis pathway and is responsible for melanocyte differentiation [48]. However, instead of A, E, and MITF‐M transcripts, we identified A, E, and a novel chimeric transcript (MITF‐novel) in the skin tissue of YTG duck with WW genotype. In contrast, the MITF‐M transcript was not detected in the skin tissue of the YTG duck (Figure 5A,D). This novel chimeric transcript fuses 151 bp 3′ end of the 3′LTR, 878 bp 5′ flanking region of the exon 2, and 230 bp original exon 2, collectively constituting the novel exon 1. Therefore, the promoter element for this novel transcript is inside the 3′LTR, and we have identified a TATA box at 6229 bp within the *Gypsy* insertion. Within the 500 bp region upstream of this promoter, we identified five putative binding sequences and three TFs (STAT3, ARID3A, SREBF1). Among them, STAT3 is capable of binding to the GAGACGGGAAA sequence, spanning from 5805 to 5815 bp of the *Gypsy* insertion.

Coding probability analysis using CPAT, PORTRAIT, and CPC2.0 revealed that the MITF‐novel transcript has a strong coding ability, supporting the existence of its protein product (Figure 5E). To further validate it, 3× Flag sequence was cloned to the N‐terminal of MITF‐novel, and western blot showed MITF‐novel is protein coding transcript. Compared to the MITF‐M transcript, MITF‐novel lacks 39 amino acids at the N‐terminal (Figure S7E). We predicted the tertiary structures to dissect the structural basis of the functional differences between MITF‐M and MITF‐novel (Figure 5F−G). The structures of MITF‐M and MITF‐novel exhibit a substantially similar conformation in the middle region of the carbon backbone (E_188_‐A_289_ of MITF‐M in marine and E_150_‐A_251_ of MITF‐novel in yellow), with a root‐mean‐square deviation value of 0.151. The structural differences were mainly concentrated in the loop regions at both N‐terminal (N) and C‐terminal (C) (Figure 5F). The N‐terminal 39 amino acids in MITF‐M form two additional stable α‐helix, S_9_‐S_21_ and M_30_‐Y_39_, around the nucleic acid binding region, according to the structure of 4ATI (*Mus musculus*) in the Protein Data Bank database [49]. As can be seen from Figure 5G, E90 and N94 in MITF‐novel form a steric hindrance in conformation, thereby affecting the binding to the nucleic acid and might further block the downstream pathway. To further determine the regulatory role of the *MITF* gene in the melanogenesis pathway, we transfected the MITF‐novel and MITF‐M transcript into DF‐1 (fibroblast cell line). Four well‐known downstream genes of the MITF‐mediated melanogenesis pathway, including tyrosinase (*TYR*), *TYRP1*, *MLANA*, and *OCA2*, were significantly downregulated in cells overexpressing the novel MITF compared to those overexpressing MITF‐M (Figure 5H). Except for the OCA2 gene, the expression level of the other three downstream genes in MITF‐novel cells showed a similar pattern to the blank control. Additionally, these four downstream genes also had almost no expression in the skin tissue of the Pekin duck (white plumage) but had significantly higher expressions in the Heiwu duck (colored plumage) (Figure S7F). This suggests that MITF‐mediated melanogenesis genes may not be activated by MITF‐novel, and the lack of TYR expression might directly cause the white plumage. The above evidence shows that the insertion of the Gypsy element induced the formation of MITF‐novel transcript and the disruption of MITF‐M, blocking the melanogenesis pathway and thus causing white plumage phenotype in ducks with the WW genotype.

### Phylogenetic analysis of *Gypsy*

**Figure 6:**
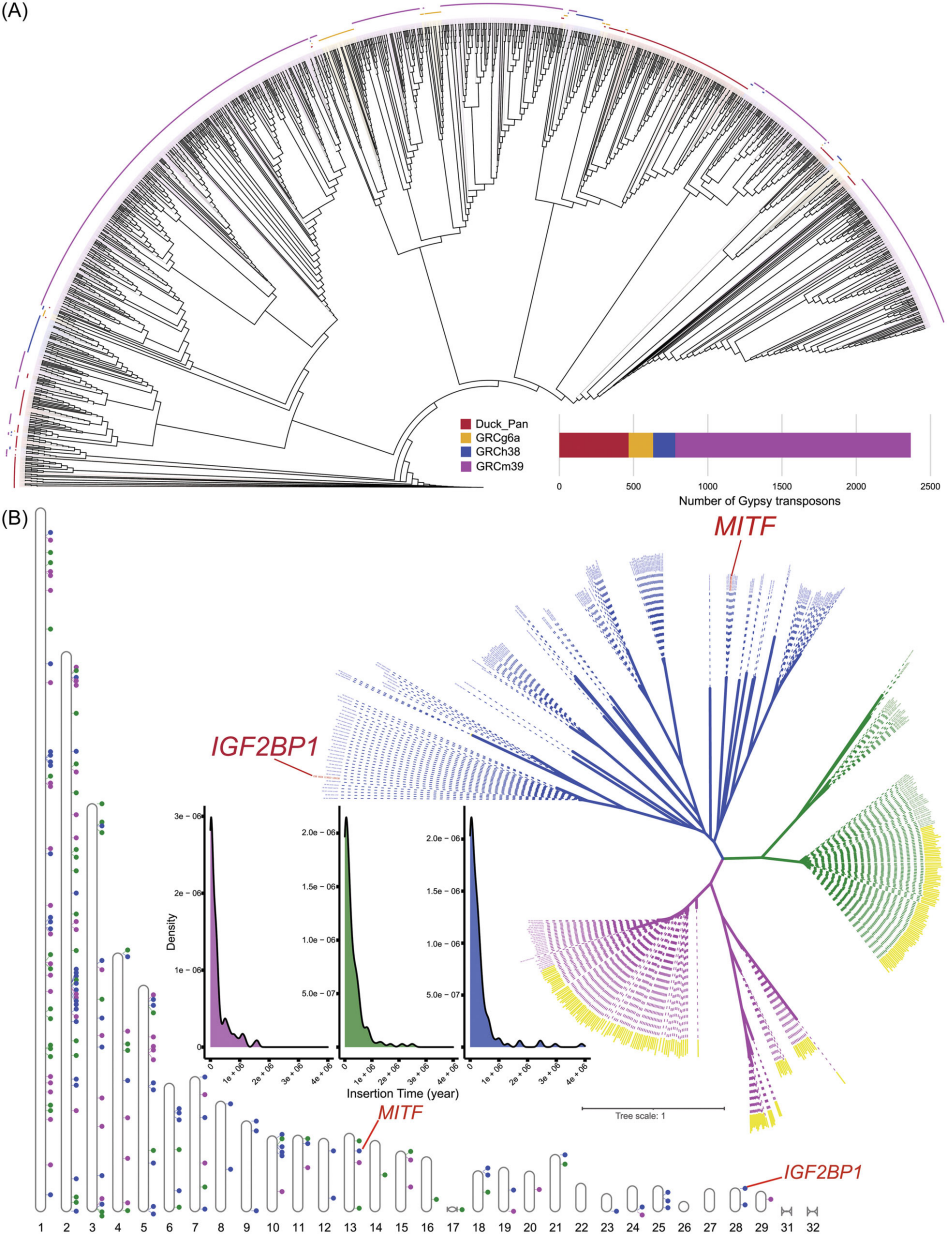
Phylogenetic analysis of LTR Gypsy superfamily and insertion time. (A) The phylogenetic tree of Gypsy transposons across the duck, chicken, human, and mouse genome. The bar chart showing the number of identified Gypsy. (B) The phylogenetic analysis of the coding region of the Gypsy superfamily located at the duck pan‐genome. The idiogram plot shows the distribution of Gypsy across the duck genome. The density plot presented the distribution of estimated insertion time for three major clades with the model of *T* = *K*/(2*r*). LTR, long terminal repeat.

The Gypsy transposon belongs to the LTR subclass of Class I retrotransposon shaping the activity and evolution of the host genome using a copy and paste approach. To determine the phylogenetic relationship of Gypsy among various species, intact Gypsy elements with two complete LTR regions were retrieved from duck, chicken, human, and mouse. Compared to chicken, an obvious expansion in the quantity of Gypsy was identified in the duck genome (467 in duck pan‐genome and 431 in ZJU1.0 Vs. 165 in chicken GRCg6a). A substantial expansion was observed in the mouse genome compared to the human genome (1586 in GRCm39 vs. 149 in GRCh38) (Figure 6A). Several relatively mixed clades were constructed, suggesting coevolution of duck Gypsy with other species. Gypsy elements in the duck genome are distributed evenly on the macrochromosome (Chr 1–6), but are primarily located on both ends of the microchromosomes (Chr 8–29) (Figure 6B). To further investigate the evolutionary origin of two Gypsy elements in duck *IGF2BP1* and *MITF*, we constructed a phylogenetic tree using the coding region sequence and the best‐fit model TVM + F + R8 based on the Bayesian information criterion scores. This tree revealed three major clades: the blue clade, which comprises Gypsy elements in *IGF2BP1* and *MITF*, is characterized by relatively long branch lengths, suggesting possibly ancient Gypsy copies. The pink and green clades, each consisting of two subclades, have relatively short branch lengths suggesting relatively recent evolutionary origin. Based on the sequence homology of the *LTR* region, we estimated insertion times of these Gypsy elements, assuming the substitution rate of 1.91 × 10^−9^ per site per year [47]. The blue clade comprised the majority of Gypsy transposons with insertion times greater than a million years, demonstrating its ancient origin (Figure 6B). Specifically, the 6945 bp Gypsy in the promoter of *IGF2BP1* and 6634 bp Gypsy in an intron of *MITF* occurred ∼3.88 and ∼0.6 million years ago, respectively, based on Sanger sequence homology. This suggests these two Gypsy transposons inserted before duck domestication (about 500 BC), which is consistent with the significantly lower frequency of the 6945 Gypsy insertion in wild mallard ducks.

## DISCUSSION

### A Gypsy element inserted into the duck *IGF2BP1* promoter carries the largest known positive effect on duck bodyweight

Duck bodyweight is a critical quantitative trait for meat production. A QTL influencing duck bodyweight was mapped near the *IGF2BP1* gene, according to SNP‐based GWAS [4]. Higher expression levels of *IGF2BP1* are linked to heavy bodyweight in duck [4] and chicken [9]. Our previous study revealed a causal deletion in the promoter of *IGF2BP1*, which could increase the bodyweight of chickens by upregulating the expression of *IGF2BP1* [9]. *IGF2BP1* knockout in mice leads to decreased bodyweight and impaired gut development [50, 51]. The current study analyzed SVs using a duck pan‐genome and revealed that a Gypsy element anchored in the *IGF2BP1* promoter region could significantly increase *IGF2BP1* transcriptional activity (Figure 4G−H). Genotype–phenotype associations demonstrated that a significant correlation between this Gypsy element and increased duck bodyweight and CW (Figure 4F), echoing the influence of the chicken *IGF2BP1*. Furthermore, this locus accounted for more than 20% of the phenotypic variance for all bodyweight and CW traits, except BW8. The highest variance explained by this locus was 27.61% for SEW. To our knowledge, the effect of this locus on bodyweight and carcass is the largest in all reported locus of ducks and even poultry. *IGF2BP1* is a N6‐methyladenosine reader that can regulate multiple biological processes, including intestinal barrier function [51], hepatic outgrowth [52], myoblast proliferation [53], adipocyte proliferation [54], axon development [55], and the abundance of microbes [56], through regulating the fate and function of target mRNA [57, 58]. These various biological processes regulated by *IGF2BP1* are all essential for body growth and development, likely contributing to the large effect of *IGF2BP1* expression on bodyweight. Collectively, this locus is the first reported functional mutation of duck *IGF2BP1* and carries the largest effect on reported bodyweight and carcass traits. The dissection of such a likely causal variant for duck bodyweight will accelerate the breeding process for meat production using marker‐assisted selection.

We have shown that this *Gypsy* element provided enhancer element which stimulate the expression of the original transcript of *IGF2BP1*. The possible involved TFs are SOX6 and SOX9, which are well‐known activator for chondrogenesis [59] and skeletal development [60]. Therefore, SOX6, SOX9, and *IGF2BP1* are highly likely co‐expressing in skeletal progenitor cells, thus the two SOX genes could interact with the 3′LTR in the H allele of *IGF2BP1*, leading to increased expression of *IGF2BP1* and larger duck body size.

### A Gypsy element anchored in the *MITF* intron contributes to the white plumage phenotype

*MITF* was previously reported as the potential causal gene associated with the white plumage trait in Pekin duck and an intronic insertion in *MITF* was linked to the MITF‐M transcript expression [4]. The MITF‐M transcript of *MITF* is exclusively expressed in melanocytes and is required for melanocyte differentiation and melanin synthesis [48]. In this study, we discovered a Gypsy insertion located in the intron of *MITF* that contributes to the white plumage phenotype in PK, YTG, and AB (Figure 5B). The potential promoter and enhancer element provided by this Gypsy element induced a novel chimeric transcript, MITF‐novel, while simultaneously inhibiting the expression of the original MITF‐M (Figure 5D). Among the putative TFs regulating this novel transcript, STAT3 is known to have a role in regulating MITF. Although STAT3 and MITF act antagonistically [61], when STAT3 expressed in the migrating and differentiating melanocyte progenitors, there is a possibility that STAT3 can bind to the Gypsy element and activate the expression of MITF‐novel. Since the A and E transcripts are also expressed in cells other than melanocytes [62], and are not affected by the Gypsy element, the white plumage trait in ducks is believed to be specific to pigmentation. This could be due to the promoter of the MITF‐novel, donated by the Gypsy element, competing with the adjacent promoter of the original MITF‐M transcript to recruit the transcriptional complex, leading to the disruption of MITF‐M transcription. In contrast, a two bp deletion in exon 11 of quail *MITF*, affecting all the transcripts, is associated with several traits, including white plumage, lower growth, lower body temperature, and a smaller heart [63]. Although the white plumage of ducks can be explained by abnormal melanin synthesis in the melanocytes, our results suggest that the direct cause of the lack of pigmentation is likely due to the absence of mature melanocytes in the feather follicles. Combining the evidence from MITF‐novel overexpressed cells and data of 1‐day old skin tissue, we analyzed the expression of four pigmentation related genes, including melanocyte progenitor markers, like *MLANA*. These genes were undetectable in the skin tissue of the Pekin duck (Figure 5H), suggesting the absence of mature melanocytes. In essence, the disruption of MITF‐M transcription, brought about by the Gypsy element insertion, likely hinders normal migration and/or differentiation of melanoblasts, indirectly leading to disruption in melanin synthesis.

### TE activity diversifies phenotypes

TE insertions can significantly impact phenotypes due to their innate structure, which includes proviral elements capable of donating promoters or enhancers that regulate transcriptional networks or generate novel transcripts [18]. However, about two‐thirds of TE insertions show weak linkage disequilibrium with adjacent SNPs [64], indicating TEs could be considered an important source of genetic variation that diversifies phenotypes. For example, a Gypsy element, undetectable using a single reference genome, inserted in the *IGF2BP1* promoter acts as an agonist to increase its transcript, leading to enlarging bodyweight. Another Gypsy element anchored in the intron of *MITF* generated a novel transcript, leading to melanin absence and white plumage formation. These two examples of TEs with significant phenotypic impacts illustrate that TE insertions can shape both quantitative and Mendelian traits. Furthermore, these two examples also support the classical evidence again that TE can affect transcriptional regulation via donating promoters and generating de novo gene birth [15, 18, 19]. Many studies have also reported on the diversifying force of TE insertions. For instance, a recent study on Norwegian sheep found that a TE insertion could suppress the expression of the beta‐carotene oxygenase 2 gene, resulting in yellow adipose tissue [65]. A LINE insertion can increase the transcriptional activity of the agouti signaling protein gene, leading to a white coat color in swamp buffalo [21]. A TE insertion in the intron 4 of *TYR* gene also induced aberrant transcripts, resulting in a recessive white plumage in chickens [66]. TE's major phenotypic impacts can also function in humans, affecting secondary palate development [22], and embryonic implantation [23]. In plants, a pan‐genome study of tomatoes revealed hundreds of SVs, most of which were TE‐related. These TEs were linked to expression changes in associated genes, potentially having significant impacts on quantitative trait variation [13]. Combining these examples and findings in our study, we suggest that TEs exert a pronounced phenotypic impact on both quantitative and Mendelian traits, affecting trait formation in livestock. Since TEs exist in almost all eukaryotic genomes, TEs can be used as additional markers to facilitate identification of causal variants for target phenotypes and further genetic improvement through genomic selection and marker‐assisted selection.

## CONCLUSIONS

In summary, we constructed the first duck pan‐genome and identified a comprehensive set of SVs in duck. A substantial number of these SVs were associated with traits related to domestication and improvement. Importantly, we dissected a Gypsy TE located at the promoter region of the *IGF2BP1*, which increased duck bodyweight by boosting its gene expression. We also revealed another Gypsy TE located within the intron of the *MITF*, which contributed to the formation of white plumage by generating a novel transcript. Our findings highlight the important impact of TEs on both quantitative and Mendelian traits, offering insights into how TEs shape phenotype formation in animals.

## METHODS

### Duck genome and genomic sequencing

The reference duck genome (ZJU1.0) [67] and four chromosome‐level duck genomes were downloaded from the National Center for Biotechnology Information and ENSEMBL database, which consists of the duckbase.refseq.v4, CAU_Pekin_2.0, CAU_Laying_1.0, and ASM874695v1 genomes [68] (Table S1). Genome quality values were assessed with Merqury [69] and showed consistency across genomes (Table S1). This study generated in‐depth genomic sequencing data of 131 unrelated duck individuals deriving from representative samples which encompassed a wide range of genetic diversity, including two wild breeds, seven Chinese indigenous breeds, and three commercial breeds (Table S2). Genomic DNA was extracted from duck peripheral blood samples using Dneasy Blood and Tissue Kit (Qiagen, #69506). DNA library was prepared according to MGISeq‐2000 library construction Protocol using MGIEasy Universal DNA Library Prep Set (BGI, # 1000006986). Generally, purified DNA is fragmented using Tn5 transposase, end‐repaired using “Reaction enhancer,” ligated using MGISeq‐2000 adapters. Libraries were treated following by purification and amplification, quantification, and circularization. Paired‐end libraries with ∼500 bp insertion size were subjected to sequencing using the MGISEQ‐2000 platform to generate paired‐end 150 bp reads (BGI Genomics Co., Ltd.).

### Pan‐genome construction

The relationships of colinear chromosomes between the four duck genomes and the reference genome were identified using the MCscan (https://github.com/tanghaibao/jcvi/wiki/MCscan-(Python-version)). We first used the *Psvcp* pipeline to construct the duck Pan‐genome.1 (Figure 1A) [26]. Briefly, query chromosome was aligned on the reference chromosome using nucmer command in mummer (v4.0.0beta2) [70] (Figure S1B), then subjected to structural variants detection using Assemblytics [71]. The insertions with a size of more than 50 bp were placed into the reference chromosome. The annotation file of reference was also updated, along with the integration of insertions. The four query duck genomes duckbase.refseq.v4, CAU_Pekin_2.0, CAU_Laying_1.0, and ASM874695v1 were aligned on the reference ZJU1.0 one by one. Second, the four query genomes were also aligned on the Pan‐genome.1 to identify the novel sequence using the *PPsPCP* pipeline [27]. The *PPsPCP* method has been developed for the construction of a linear pan‐genome using multiple genomes. This is achieved by identifying novel genomic sequences in the query genome that are absent from the reference genome via alignment and then appending these genomic sequences as the additional new sequences to the end of the reference genome. The *Psvcp* is robust at capturing and localizing insertions present in other genomes but absent in the backbone reference genome. However, it does not effectively handle other forms of SVs like substitutions, and tandem repeat contractions and expansions, which are proficiently captured by *PPsPCP*. By amalgamating both the *Psvcp* and *PPsPCP* methods, we can maximize the extension of the reference genome by incorporating these novel sequences and capturing more complex SVs. Therefore, we combined two pipelines to construct the duck linear pan‐genome.

All novel contigs were merged and then redundant assembled sequences were filtered using CD‐HIT [72] (‐c 0.9 ‐aS 0.8 ‐d 0 ‐sf 1) with the threshold of 90% similarity. New contigs of non‐reference sequences with lengths larger than 500 bp were kept. Novel contigs were aligned using blastn (v2.9.0) [73] against the NT database (v5, 07‐03‐2019) of contaminant taxid groups, which includes archaea, viruses, bacteria, fungi, and Viridiplantae to identify the contaminant sequences. However, we could not find any hits with identities larger than 90% and query lengths larger than 50%. The final contamination‐free non‐reference sequences and the Pan‐genome.1 were merged to generate the duck pan‐genome. The novel contigs were annotated with ASM874695v1 annotation file using the GMAP(v2021‐08‐25) [74].

### PAV calling and pan‐genome modeling

Steps for PAV calling was described in our previous study [9]. Briefly, the longest transcripts of each gene were retrieved, and coding sequence regions were extracted. Genes that cumulative coverage of at least two reads with more than 5% of all exons was considered as presence, otherwise absence [75]. Clean reads were aligned to the duck pan‐genome using BWA‐MEM (v0.7.17) [76] and the sequencing depth was counted using Mosdepth package (v0.2.5) [77]. It is confirmed that the sequencing data with more than 10× in each depth was allowed to obtain a PAV matrix with a 99.4% accuracy rate [9]. In this study, the average depth of all sequencing data was more than 45×, which increased the robustness of PAV calling. A pan‐genome curve was constructed using a power‐law regression: *y* = *A* × *B* + *C* to assess whether the duck genomes are sufficient to represent the genetic diversity of the duck. A core genome curve was performed using an exponential regression model: *y* = *AeBx* + *C*. In these two equations, *y* was the total number of the gene, *x* was the genome number, and *A*, *B*, and *C* were the fitting parameters.

### SV calling

We first constructed a duck pan‐genome by combining the *Psvcp* and *PPsPCP* pipeline, using four public genomes and the reference genome, and then identified the SVs using high‐depth population‐wide WGS data. This methodology has two primary advantages for SV discovery. First, our combined approach allows for the placement of insertions from the query genomes into the reference genome, resulting in a chimeric pan‐genome. This chimeric pan‐genome integrates novel sequences absent from the reference genome, facilitating the identification of novel SVs, as insertions are often difficult to identify using short‐read data. Second, the high‐depth population‐scale WGS data, together with a pan‐genome, can improve the SV discovery as the pan‐genome is derived from representative individuals. To facilitate the accurate alignment of sequencing reads at the boundaries of novel contigs, 200 bp flanking sequences on either side were added [78]. High‐depth sequencing data was mapped on the duck pan‐genome with flanking sequencing. Bam files were sorted, and duplicate reads were removed using Sambamba (v0.8.2) [79]. Structural variants were called using four detecting tools including LUMPY (v0.2.13) [30], Delly (v0.8.7) [31], GRIDSS (v 2.1.0) [32], and Manta (v1.6.0) [33]. LUMPY generated deletions, which were less than 340 bp and no split read support, were filtered to reduce the false calls [35]. Subsequently, the VCF files were genotyped using SVTyper (v0.0.4) [80]. We used Delly, GRIDSS, and Manta to identify and genotype the SVs with default parameters. SV genotypes from four tools were merged and filtered using SURVIVOR [81] with the parameters “SURVIVOR merge 1000 2 1 1 0 50” to increase the robustness.

### SV analysis

Population genetic analysis was conducted using the binary SV genotype. Biallelic SVs located on autosomes were retained and subjected to filter against the variants with MAF < 0.01 using PLINK (v1.9) [82]. A phylogenetic tree was constructed using the IQ‐TREE software (v1.6.12) [83] with 1000 bootstrap replicates based on the GTR model and visualized using the iTOL online web server [84]. PCA was implemented using smtpca of the EIGENSOFT [85]. Population assignment analysis was conducted using the Admixture software [86].

To identify the SVs with significantly changed occurrence frequency during domestication or improvement, the derived allele frequencies were compared between the native breeds and wild breeds or commercial breeds. The wild group comprised Mallard duck and Chinese‐spot‐billed duck, while the commercial group consisted of Pekin duck, Cherry Valley duck, and Grimaud freres duck. The other seven populations were indigenous duck breeds, defined as the native group. The significance of variations in SV frequencies between groups were ascertained by intersecting the results of Fisher's exact test with a FDR of 0.001 [9, 37] and Weir and Cockerham's *F*_ST_, using the 99th percentile threshold value as computed with Vcftools [87]. Significantly increased SVs during domestication or improvement, were defined as SVs having a significantly higher frequency in native breeds than wild breeds, or commercial breeds than native breeds, respectively. Inversely, we consider SVs with a significantly lower frequency as significantly decreased SVs.

### Detection of TE detection and their association with SVs

TEs can be classified into two classes: DNA transposons and retrotransposons, depending on genetic structures and transposition mechanisms. LTRs, long interspersed nuclear elements (LINEs), and short interspersed nuclear elements are the dominant retrotransposons in vertebrates [19]. Interspersed repeats and low complexity DNA sequences across the duck pan‐genome were screened using RepeatMasker (v4.0.8) [88]. A custom repeat library was constructed using RepeatModeler (v1.0.11) [89]. Repeat sequences detected based on the custom repeat library and one the RepBase database (downloaded in June 2019) of vertebrates were scanned separately and merged using ProcessRepeats program of RepeatMasker. Abundance, length, and divergence rate were abstracted from the result file of RepeatMasker (v4.0.8) [88]. TE families with copy numbers more than 2000 were collected to construct the classification tree. The top 17 class/superfamilies filtered according to their copy numbers were retrieved to perform the statistics of length, abundance, and divergence levels. To examine the occurrence correlation between SVs and TEs, we divided the genome into windows with 2, 5, and 10 kb and subject to screen the presence or absence of TE and SV. The occurrence matrix was subjected to the *χ* ^2^ test using the R package “ggstatsplot” [90].

### Identification of intact TEs and estimation of insertion time

Intact TEs were detected using EDTA [91] with default parameter. Structural and proviral elements were resolved by RetroTector [92] and EDTA software. Insertion time of LTR TEs was estimated with the model of *T* = *K*/(2*r*) (https://github.com/wangziwei08/LTR-insertion-time-estimation), assuming that the substitution rate is 1.91 × 10^−9^ per site per year [47].

### GO annotation

To identify the genes affected by SVs, bedtools (v2.29.2) [93] was used to search for genes physically closest to SVs. Functional annotation of the duck pan‐genome was performed using the command line Blast2GO (v2.5) [94]. The longest transcript of each gene was retrieved from the pan‐genome and subjected to alignment to the proteins in the Uniref90 database (downloaded on March 2022) using BLASTP function in Diamond [95] with the threshold *E* < 1 × 10^−3^. GO annotation of these genes was conducted by the R package topGO [96] using Fisher's exact test with the approach “elim” for multiple comparisons correction.

### Calculation of absolute △AF

Of the WGS data from 131 duck individuals used in this study, 41 are from commercial meat breeds (Pekin, Cherry Valley, and Grimaud freres ducks) with high body weight and thus categorized into the high group, and other 90 individuals were categorized into the low group. Variants located within the 10 kb upstream and 10 kb downstream regions (pan‐genome chr28:103034−130138) of the *Gypsy* element were extracted. The △AF value for each variation (SNPs and SV) was calculated by the comparison of allele frequencies between the two groups.

### Genotyping of SVs and association analysis

Primers were designed to genotype the SVs based on the flanking sequence (Table S7). PCR genotyping was described as our previous study [9]. A general linear model was conducted to investigate the association between *IGF2BP1* genotypes and phenotypes using TASSEL5 software with sex factor defined as a fixed effect [97]. The value of marker *R* ^2^ was determined to explain the phenotypic variation derived from genotypes, which was computed from the marker sum of squares after fitting all other model terms divided by the total sum of squares. The White Liancheng × White Kaiya Cross F2 population was established at Hankou Jingwu Food Industry Garden Ltd. All ducks were hatched on the same day and raised in cages in a semipen house under standard management conditions. Bodyweight was recorded at 8 or 9 weeks of age (referred to as BW8 and BW9). At slaughter, individual measurements were taken for carcass BWHR, both EW and SEW, LW, BMW, and postprocessing CW using a scale.

### Functional assay of *IGF2BP1* and *MITF*

RACE was conducted to amplify the full length of *IGF2BP1* and *MITF* transcripts using SMARTer Race 5′/3′ Kit with gene specific primers (Table S8). To further verify the molecular effects of the insertion, luciferase expression levels were investigated to represent the transcriptional activity through transfecting two kinds of recombinant plasmids (pGL3‐L and pGL3‐H). Promoter region of *IGF2BP1* (i.e., the 2146 bp sequence between the *Gypsy* inserted site and the original TSS of *IGF2BP1* for pGL3‐L; the 9,250 bp sequencing including the entire *Gypsy* insertion and the sequence between *Gypsy* and TSS of *IGF2BP1* for pGL3‐H) was cloned into the pGL3‐Basic luciferase vector (Promega) that was subjected to transfection into DF‐1 cell line (chicken fibroblast cell) together with PRL‐TK plasmid. Transcriptional activity was investigated by Dual‐Luciferase Reporter Assay System (Promega) after 48 h of transcription. The mRNA level of *IGF2BP1* and *MITF* were determined by quantitative PCR with their specific premiers (Table S7), normalized by *GAPDH* gene using the $2^{‐∆∆C_{t}}$ method. AlphaFold2 was used to predict different conformational structures for MITF‐M and MITF‐novel [98, 99]. Protein structural analysis and all colored schemes are accomplished by PyMOL 2.5. The 3 × FLAG sequence was inserted into the N‐terminal of the MITF‐M and MITF‐novel transcripts and then transfected into the DF‐1 cell line. After 48 h of transfection, their regulatory effects on the four well‐known downstream genes in the MITF‐mediated melanogenesis pathway were determined using the quantitative PCR (Table S8). Evolutionary analysis on IGF2BP1 and MITF was performed using Vcftools. A 20 kb window with 20 kb step was set in computing which F_ST_ and Π statistics, while a 10 kb window with 5 kb step for Tajima's D indicator.

### RNA‐Seq analysis

Transcriptome data of liver and sebum from mallard and pekin at 2, 4, and 6 weeks of age was downloaded from the National Genomics Data Center (China National Center for Bioinformation) from the project number PRJCA002795, PRJCA002803, PRJCA002808, and PRJCA002807 [68]. Transcriptome data of the skin tissue was downloaded from project number PRJCA003516 [100]. Raw reads were filtered using Trimmomatic (v 0.39) [101] and subjected to align the duck pan‐genome using STAR (v2.7.1a) [102]. Expression levels of transcripts were determined using featureCounts (v2.0.0) [103]. These RNA‐Seq data was categorized into 25 groups (*n* = 6 in each group) based on shared tissue and developmental stage. The average fragments per kilobase per million (FPKM) for each of the liver, fat, and leg muscle groups for each gene were calculated and the gene with the minimal group average FPKM greater than 3 were defined as expressed gene which were used for searching for putative TFs for *IGF2BP1*. For *MITF*, skin and hair follicle tissues were applied while the cut off for expressed gene was that minimal group average FPKM greater than 6.

### Predictions of putative TFs

Putative TF binding sites were predicted by the online JASPAR TF database [104]. All 2430 TFs in the database were selected for scanning their binding sites in the 3′LTR sequence of the 2 *Gypsy* insertion. The threshold of relative score of binding was set as 0.8 and reverse strand predicted sequences were excluded. The predicted TFs were searched for in the list of expressed genes based on the RNA‐Seq data.

## AUTHOR CONTRIBUTIONS

Wenting Li, Kejun Wang, Xiangtao Kang, and Haifei Hu conceived the project and designed research. Kejun Wang and Wenting Li designed the analysis and wrote the manuscript. Kejun Wang, Wenting Li, Haifei Hu, Jingyi Li, and Armin Scheben revised the manuscript. Kejun Wang, Guoying Hua, Jingyi Li, and Yanan Wu performed analysis. Wenting Li, Chenxi Zhang, Lan Yang, and Xiaoyu Hu performed the wet‐lab experiment. Yu Yang, Ping Gong, Kejun Wang, Yanzhang Gong, Shuangjie Zhang, Yanfeng Fan, Tao Zeng, Lizhi Lu, Xiangtao Kang, Yadong Tian, Guirong Sun, and Ruirui Jiang contributed to sample collection and construction of F2 resource population.

## CONFLICT OF INTEREST STATEMENT

The authors declare no conflict of interest.

## ETHICS STATEMENT

Ethics approval (HNND2023103002) for this study was approved by Henan Agricultural University.

## Supporting information


**Figure S1**. Genome phylogenetic distance (A) and colinear analysis between four duck assemblies and reference genome (ZJU1.0) (B).
**Figure S2**. GO enrichment analysis of dispensable genes in duck pan‐genome.
**Figure S3**. SV distribution and population structure.
**Figure S4**. Population structure constructed by whole‐genome SNPs.
**Figure S5**. Transposable elements (TEs) annotation across the duck pan‐genome.
**Figure S6**. Comparison of allele and expression level between genotypes for the insertion in duck *IGF2BP1* promoter
**Figure S7**. Comparison of alleles, amino acid sequences and expression levels between genotypes for the insertion in the intron of duck *MITF*.

**Table S1**. Duck genomes used for duck pan‐genome construction.
**Table S2**. Statistics summary of duck reference genome and pan‐genome.
**Table S3**. Summary of whole genome sequencing data used for SV calling.
**Table S4**. SV validation.
**Table S5**. Gene list of significant SV during domestication.
**Table S6**. Gene list of significant SV during improvement.
**Table S7**. List of intact TE‐derived SVs across the duck pan‐genome.
**Table S8**. Primers used in this study.

## Data Availability

All the sequence data generated in this study have been deposited in the National Genomics Data center (https://bigd.big.ac.cn) with the accession codes PRJCA011446. The duck pan‐genome and relevant data are available in the DRYAD database (https://doi.org/10.5061/dryad.wwpzgmsnt). The data and scripts used are saved in GitHub https://github.com/liwenting5959/Duck_pan-genome. Supporting Information: Materials (figures, tables, scripts, graphical abstract, slides, videos, Chinese translated version, and update materials) may be found in the online DOI or iMeta Science http://www.imeta.science/.
