# Supplementary material for: Duck pan‐genome reveals two transposon insertions caused bodyweight enlarging and white plumage phenotype formation during evolution
Source: Imeta. 2023 Dec 17;3(1):e154. doi: 10.1002/imt2.154 (PMC10989122; doi:10.1002/imt2.154)
Supplement: Supplementary file 1 — Figure S1. Genome phylogenetic distance (A) and colinear analysis between four duck assemblies and reference genome (ZJU1.0) (B). Figure S2. GO enrichment analysis of dispensable genes in duck pan‐genome. Figure S3. SV distribution and population structure. Figure S4. Population structure constructed by whole‐genome SNPs. Figure S5. Transposable elements (TEs) annotation across the duck pan‐genome. Figure S6. Comparison of allele and expression level between genotypes for the insertion in duck IGF2BP1 promoter Figure S7. Comparison of alleles, amino acid sequences and expression levels between genotypes for the insertion in the intron of duck MITF. [file IMT2-3-e154-s002.docx]

**Supporting information to:**

**Duck pan-genome reveals two transposon insertions caused bodyweight enlarging and white plumage phenotype formation during evolution**

**Running title:**

**Duck pan-genome reveals diversifying effect of two TE insertions on phenotype**

Kejun Wang^1,2^**^#^**, Guoying Hua^3^**^#^**, Jingyi Li^4^**^#^**, Yu Yang^5^**^#^**, Chenxi Zhang^1,2^, Lan Yang^1,2^, Xiaoyu Hu^1,2^, Armin Scheben^6^, Yanan Wu^7,8^, Ping Gong^5^, Shuangjie Zhang^9^, Yanfeng Fan^9^, Tao Zeng^10^, Lizhi Lu^10^, Yanzhang Gong^4^, Ruirui Jiang^1,2^, Guirong Sun^1,2^, Yadong Tian^1,2^, Xiangtao Kang^1,2*^, Haifei Hu^11*^, Wenting Li^1,2*^

^1^Henan Key laboratory for innovation and utilization of chicken germplasm resources, College of Animal Science and Technology, Henan Agricultural University, Zhengzhou 450046, China

^2^ The Shennong Laboratory, Zhengzhou 450002, China

^3^Agricultural Genomics Institute at Shenzhen, Chinese Academy of Agricultural Sciences, Shenzhen, 518120, China

^4^Key Laboratory of Agricultural Animal Genetics, Breeding and Reproduction of Ministry of Education, College of Animal Science and Technology, Huazhong Agricultural University, Wuhan 430070, China

^5^Wuhan Academy of Agricultural Science, Wuhan 430070, China

^6^Simons Center for Quantitative Biology, Cold Spring Harbor Laboratory, Cold Spring Harbor, NY, USA

^7^College of Veterinary Medicine, Henan Agricultural University, Zhengzhou 450046, China

^8^International Joint Research Center for National Animal Immunology, Zhengzhou 450046 Henan, China

^9^Jiangsu Institute of Poultry Sciences, Yangzhou 225125, China

^10^State Key Laboratory for Managing Biotic and Chemical Threats to the Quality and Safety of Agro-Products, Institute of Animal Husbandry and Veterinary Science, Zhejiang Academy of Agricultural Sciences, Hangzhou 310021, China.

^11^Rice Research Institute & Guangdong Key Laboratory of New Technology in Rice Breeding & Guangdong Rice Engineering Laboratory, Guangdong Academy of Agricultural Sciences, Guangzhou 510640, China.

**^#^** These authors contributed equally: Kejun Wang, Guoying Hua, Jingyi Li, Yu Yang

^*^Correspondence: [liwenting_5959@hotmail.com](mailto:liwenting_5959@hotmail.com) (Wenting Li), [huhaifei@gdaas.cn](mailto:huhaifei@gdaas.cn) (Haifei Hu), [xtkang2001@263.net](mailto:xtkang2001@263.net) (Xiangtao Kang)

**
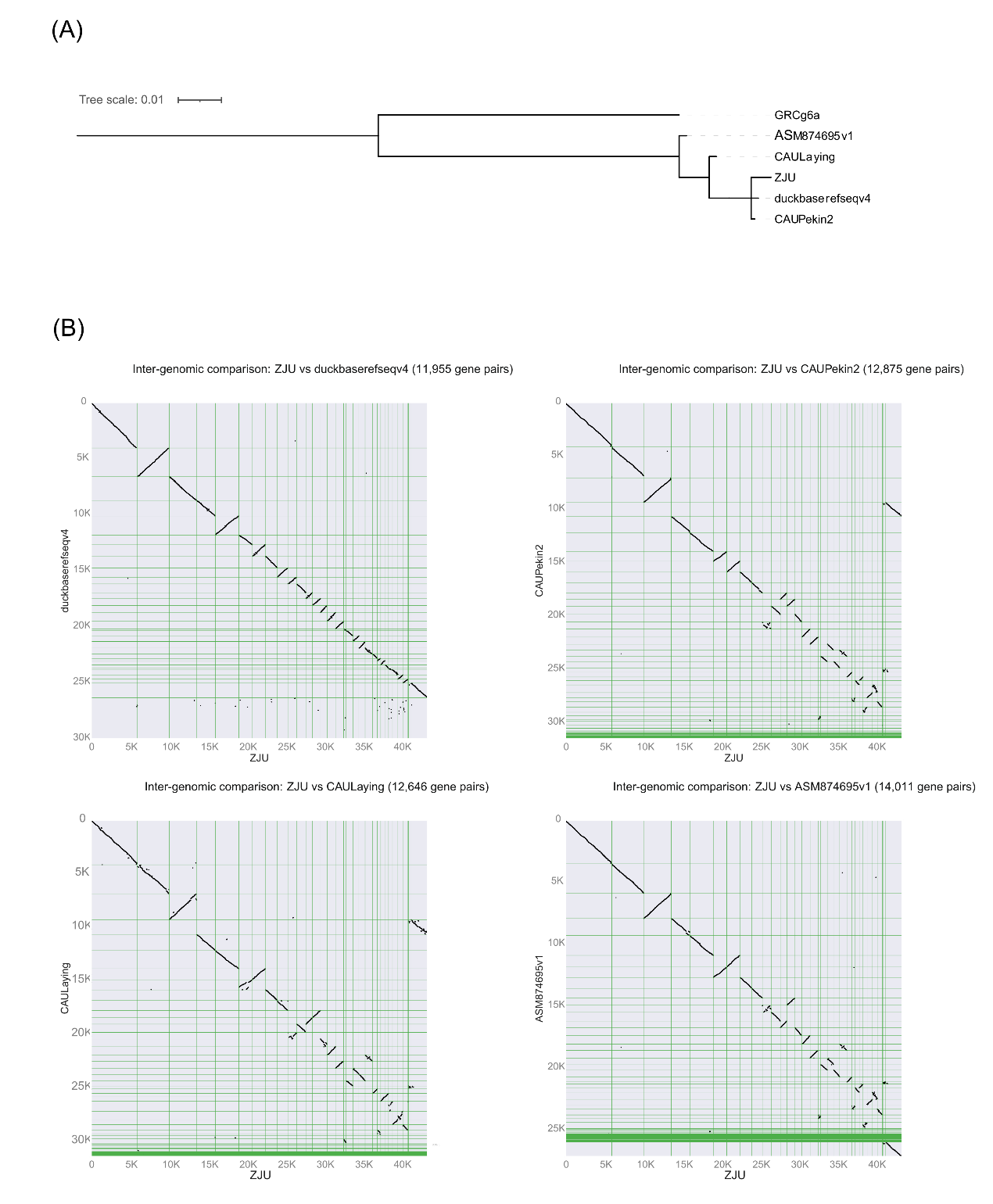
**

**Figure S1.**  Genome phylogenetic distance (A) and colinear analysis between four duck assemblies and reference genome (ZJU1.0) (B).


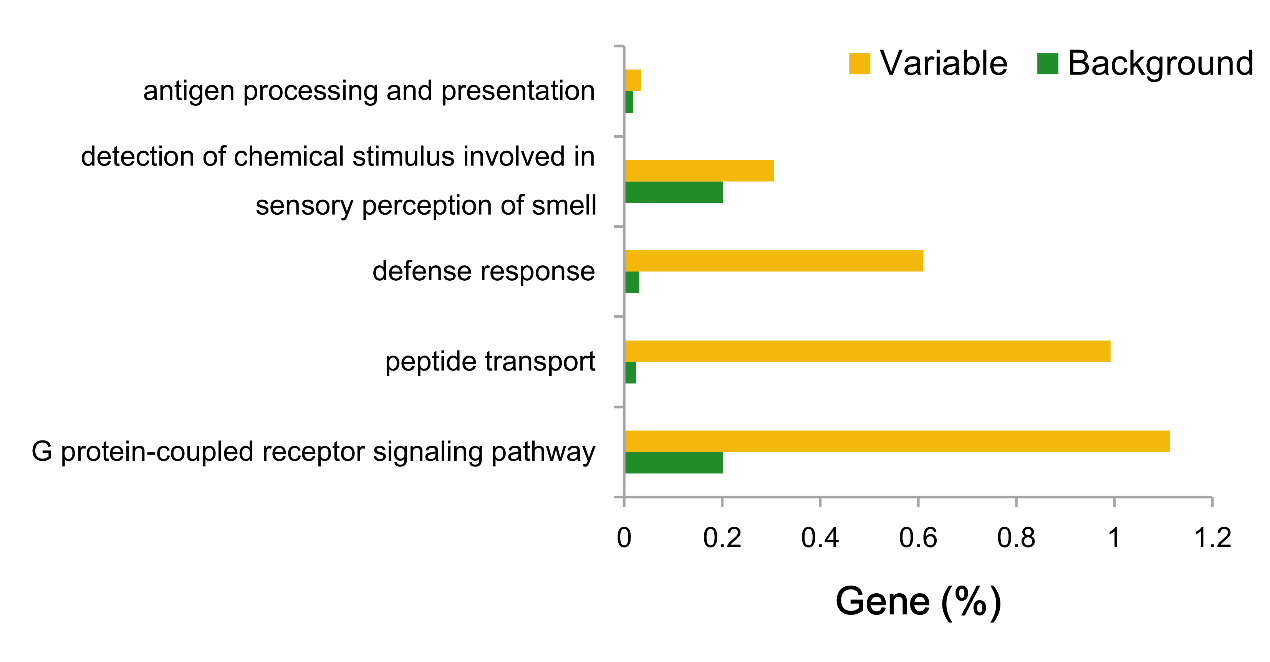


**Figure S2.** GO enrichment analysis of dispensable genes in duck pan-genome.

**
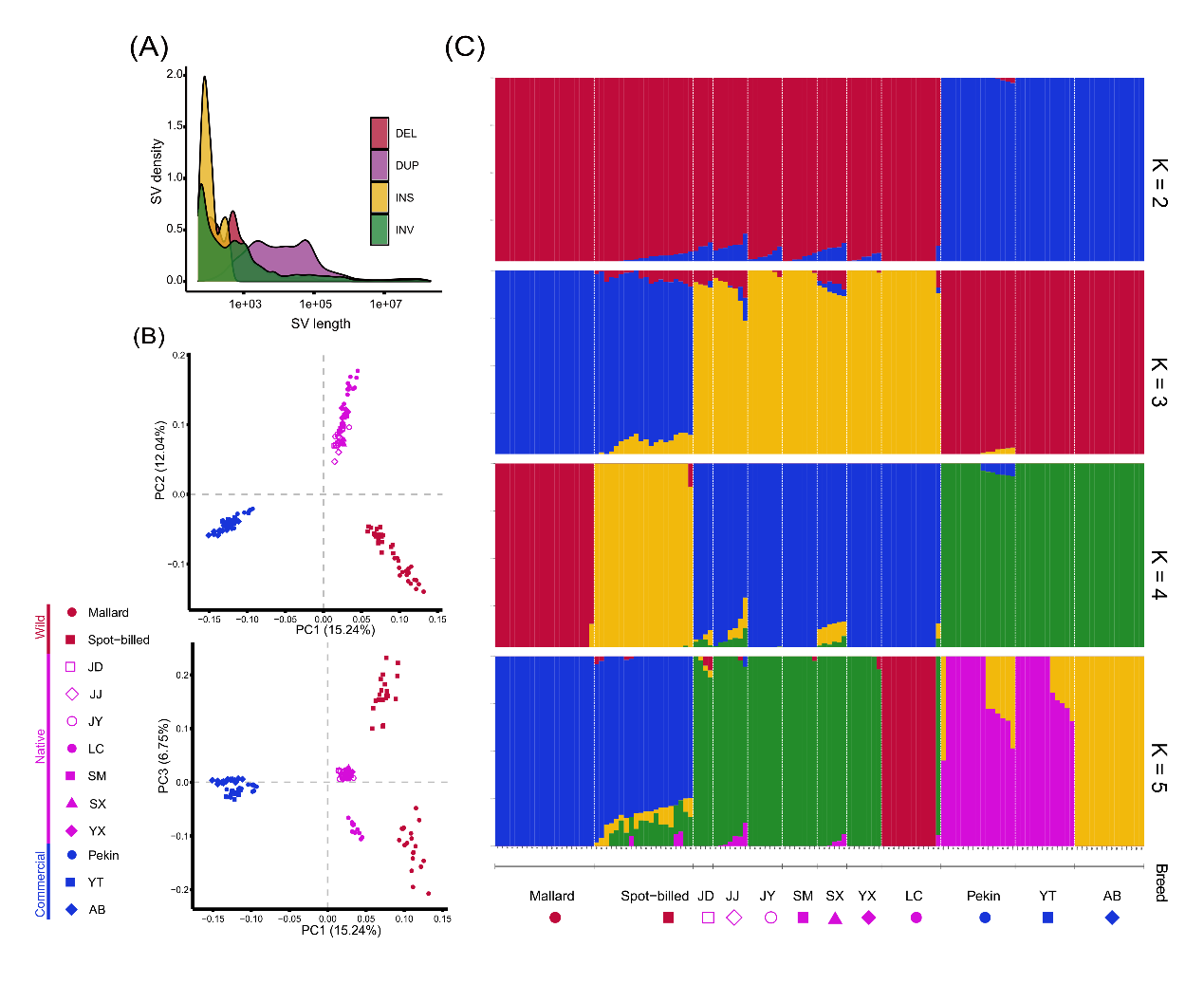
**

**Figure S3.** SV distribution and population structure. (A) Length distribution of identified SVs using pan-genome. (B)The principal component analysis (PCA) of duck breeds based on SVs with (upper) PC1 and PC2 or (bottom) PC3. The breed abbreviations are as in Figure 1. (C) Population structure with different numbers of ancestral kinships (K = 2, 3, 4, and 5) based on SVs.


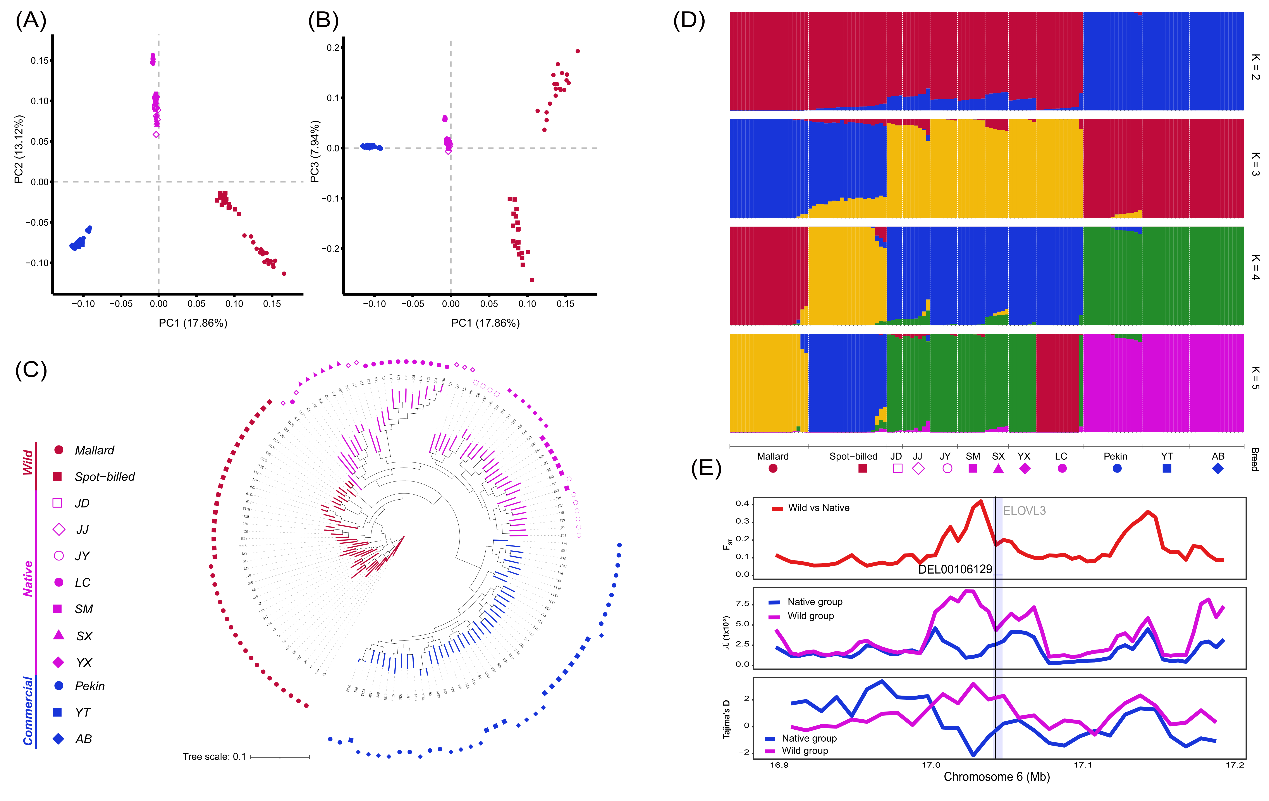


**Figure S4.** Population structure constructed by whole-genome SNPs. The principal component analysis of duck breeds based on SNPs with PC1 and PC2 (A) or PC3(B). The breed abbreviations are as in Figure 1. (C) Phylogenetic tree constructed based on SNPs with GTR model. (D) Population structure with different numbers of ancestral kinships (K = 2, 3, 4, and 5) based on SNPs. (E) SNP-based selective sweep analysis on DEL00106129 SV between native group and wild group.


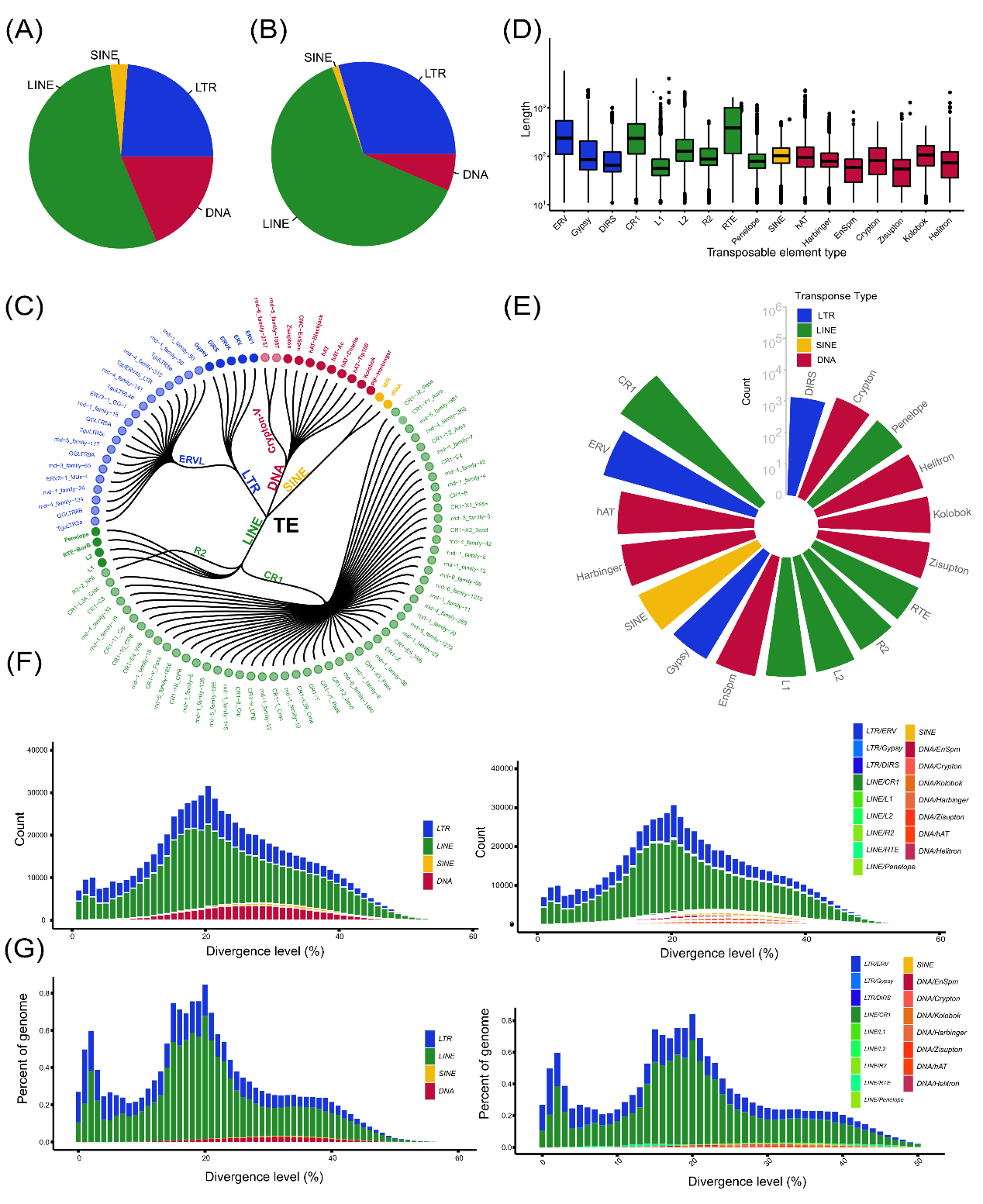


**Figure S5.** Transposable elements (TEs) annotation across the duck pan-genome. The proportions of TEs from different classes in the (A) count and (B) length. (C) Classification of duck TEs classes, clades/superfamilies, and families with copies of more than 2000. Solid circles represent the superfamilies, while light solid circles represent the families. Distributions of (D) length and (E) count of 17 most prevalent TE clades/superfamilies. Sequence divergence distributions of TE class in (F) count and genome (G) coverage.


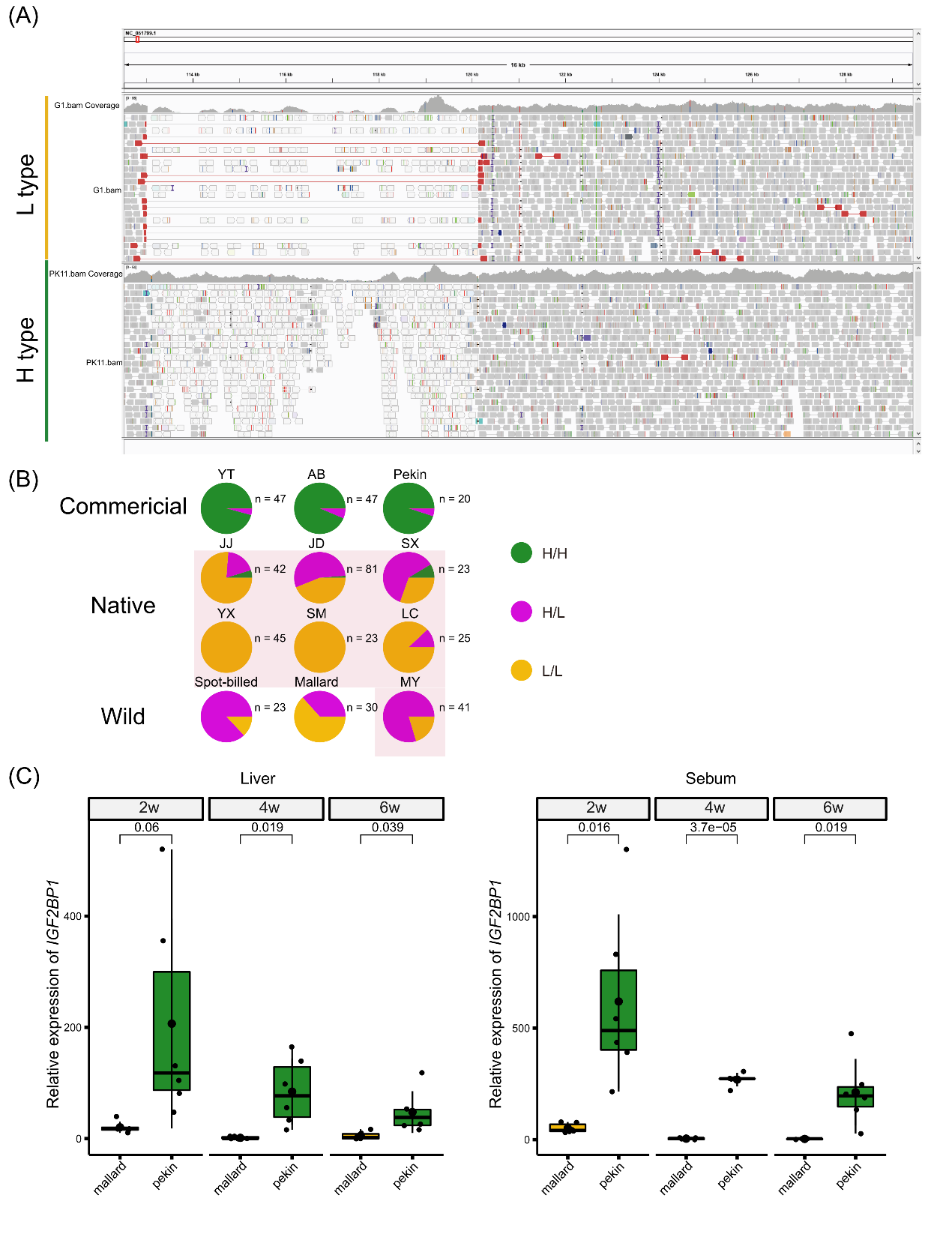


**Figure S6.** Comparison of allele and expression level between genotypes for the insertion in duck IGF2BP1 promoter. (A) Read alignment for the regulatory region of IGF2BP1 visualized using Integrative Genomics Viewer. (B) Genotypic frequencies of IGF2BP1 promoter insertion. (C) expression level of IGF2BP1 between mallard (H/H) and pekin (L/L) at 2, 4 and 6 weeks of age.


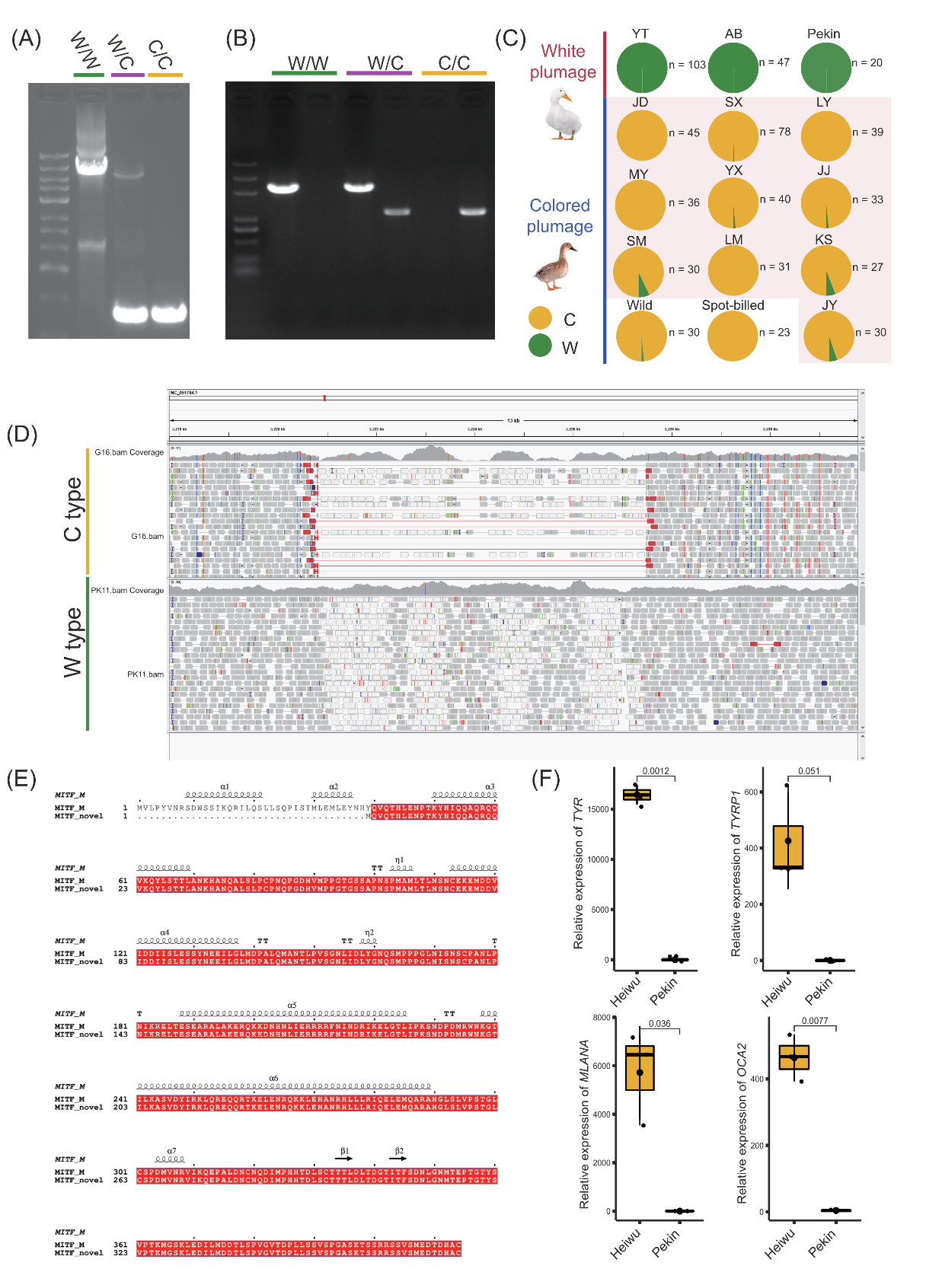


**Figure S7.** Comparison of alleles, amino acid sequences and expression levels between genotypes for the insertion in the intron of duck MITF. Gel plots for (A) PCR genotyping, (B) allelic-specific PCR genotyping and (C) allelic frequencies in validating population with white and colored plumage. (D) Read alignments for the intron of MITF visualized using Integrative Genomics Viewer. (E) Amino acid sequence alignment between MITF-M and MITF-novel transcripts. (F) RNA-seq results showing the expression levels of four downstream genes of MITF involved in the melanogenesis pathway in skin tissues of colored plumage duck (Heiwu duck) and white plumage duck (Pekin duck).
